# The contribution of inhibitory interneurons to circuit dysfunction in Fragile X Syndrome

**DOI:** 10.3389/fncel.2014.00245

**Published:** 2014-08-25

**Authors:** Christian A. Cea-Del Rio, Molly M. Huntsman

**Affiliations:** ^1^Department of Pharmaceutical Sciences, Skaggs School of Pharmacy and Pharmaceutical Sciences, University of Colorado, Anschutz Medical CampusAurora, CO, USA; ^2^Department of Pediatrics, School of Medicine, University of Colorado, Anschutz Medical CampusAurora, CO, USA

**Keywords:** GABA, synchronization, inhibitory neurotransmission, synaptic transmission, interneurons

## Abstract

Many neurological disorders, including neurodevelopmental disorders, report hypersynchrony of neuronal networks. These alterations in neuronal synchronization suggest a link to the function of inhibitory interneurons. In Fragile X Syndrome (FXS), it has been reported that altered synchronization may underlie hyperexcitability, cognitive dysfunction and provide a link to the increased incidence of epileptic seizures. Therefore, understanding the roles of inhibitory interneurons and how they control neuronal networks is of great importance in studying neurodevelopmental disorders such as FXS. Here, we present a review of how interneuron populations and inhibition are important contributors to the loss of excitatory/inhibitory balance seen in hypersynchronous and hyperexcitable networks from neurodevelopmental disorders, and specifically in FXS.

## Introduction

Fragile X Syndrome (FXS) is one of several disorders associated with autism spectrum disorders (ASDs)—a heterogeneous group of behaviorally identified neurodevelopmental disabilities. The prevalence rate of autism in FXS reportedly ranges from 25% to 52% (Kaufmann et al., 2004; García-Nonell et al., 2008; Hall et al., 2008), often presenting ASD features such as social avoidance (Marco and Skuse, 2006). Also, FXS is the most common inherited cause of intellectual disability with an average IQ of 40 (Merenstein et al., 1996). Because of its association to the X chromosome, FXS has a higher prevalence in males as approximately of 1 in 3600–4000, than females (approximately 1 in 4000–6000) (Coffee et al., 2009). FXS is attributed to the transcriptional silencing of the Fragile X Mental Retardation 1 (*FMR1*) gene and the consequent loss of the gene product of *FMR1—*Fragile X Mental Retardation Protein (FMRP; Penagarikano et al., 2007). In the human condition the silencing of *FMR1* is caused by hypermethylation—this occurs when a trinucleotide (CGG) repeat located in the 5’ untranslated region of the gene expands to a length of more than 200 repeats. The loss of this protein is far reaching because FMRP interacts with approximately 4–8% of all synaptic mRNAs and regulates the translation of numerous synaptic proteins and receptor systems (Brown et al., 2001).

The FXS phenotype involves hyperactivity, attention deficits, poor eye contact, shyness, self-talk, anxiety, mood instability, hyperarousal to sensory stimuli, and autism (Hagerman and Hagerman, 2002). Defects underlying neurodevelopmental disorders, including FXS, are widely believed to lie at the level of the synapse (Zoghbi, 2003; Ebert and Greenberg, 2013). In FXS, these profound changes include alterations in both excitatory and inhibitory neurotransmission across multiple brain regions (Huber et al., 2002; Bear et al., 2004; Bureau et al., 2008; Harlow et al., 2010; Olmos-Serrano et al., 2010; Till et al., 2012; Van der Molen et al., 2012; Kim et al., 2013). Although excitatory/inhibitory balance has been a recent subject of study in FXS research, not much is known of how interneuron populations contribute to the phenotype. In this review, we summarize current knowledge of FXS behavioral and cognitive phenotype, the circuitry abnormalities related to them and how interneurons are an important subject of study to understand alterations in neuronal networks.

## Cognition and behavioral processing in FXS

Since the *FMR1* gene was first identified and linked to FXS in 1991 (Verkerk et al., 1991), tremendous progress has been made to understand the neurological deficits that contribute to the phenotype. Most of the cognition and behavioral abnormalities have been investigated to try to understand how FMRP is involved in the neurobiological processing of brain areas related to these specific tasks. For instance, lack of FMRP found in the mouse model of FXS leads to cerebellar deficits at both the cellular and behavioral levels and raise the possibility that cerebellar dysfunctions can contribute to motor learning deficits in FXS patients (Koekkoek et al., 2005). Indeed, although premutation carriers of FMRP lead to a different syndrome (FXTAS), they showed an absence of cerebellar inhibition over primary motor cortex and a reduced GABA-mediated intracortical and afferent inhibition compared with healthy individuals (Conde et al., 2013) that could potentially also be present in FXS patients. Moreover, FXS patients display specific emotion recognition deficits for angry and neutral (but not happy or fearful) facial expressions through visual scanning tasks (Shaw and Porter, 2013), that in turn is directly related to formation and function of neuronal circuits attributed to behavioral processes such as fear, emotion recognition and anxiety carried out by the amygdala (Olmos-Serrano and Corbin, 2011; Kim et al., 2014). These socio-emotional deficits are also associated with deficits in neuronal processing of sensory systems. Studies have shown that together with a shift change in development for synaptic formation and plasticity in the amygdala (Kratovac and Corbin, 2013; Vislay et al., 2013), impaired critical plasticity periods for auditory, visual and somatosensory cortex also occurred in FXS (Bureau et al., 2008; Harlow et al., 2010; Till et al., 2012; Van der Molen et al., 2012; Kim et al., 2013). Therefore these studies reveal a role for FMRP in shaping sensory circuits during developmental critical periods when time windows of protein expression are vulnerable to alterations (reviewed in Meredith et al., 2012). Dendritic spine stability, branching and density abnormalities are part of the developmental delay observed in these same brain areas (Cruz-Martín et al., 2010; Pan et al., 2010; Till et al., 2012; Lauterborn et al., 2013) and they depend on the environmental context and experience that they are undergoing. Other characteristics of cortical neuronal networks in FXS are hyperesponsivness and hyperexcitability (Gonçalves et al., 2013; Rotschafer and Razak, 2013), making these circuits highly synchronous which taken together suggest excitatory/inhibitory balance abnormalities of the FXS neuronal circuitry. These state-dependent network defects could explain the intellectual and sensory integration dysfunctions associated with FXS.

## Excitatory/inhibitory balance in FXS neuronal networks

FXS neuronal networks are hyperexcitable (Gibson et al., 2008; Olmos-Serrano et al., 2010; Gonçalves et al., 2013; Rotschafer and Razak, 2013). This explains why most studies focus on excessive excitatory activity. The majority of research about excitatory drive and synaptic plasticity that describes hyperexcitability in FXS is illustrated in the “mGluR theory” (Huber et al., 2002; Bear et al., 2004). Briefly, the mGluR theory explains that the psychiatric and neurological aspects of FXS are a consequence of exaggerated responses to metabotropic glutamate receptor (mGluR) activation (Huber et al., 2002). One response is mediated by a synaptic plasticity process known as long term depression (LTD; Huber et al., 2002; Bear et al., 2004). Additional studies also reveal that pharmacological intervention of mGluR activation can rescue the FXS phenotype in the *Fmr1* mouse model suggesting a therapeutic role for inhibitors of mGluR activity- specifically type 1 and type 5 receptor activity (Dölen et al., 2007; Michalon et al., 2012; Ronesi et al., 2012). Due to initial early success of 2-methyl-6-(phenylethynyl)pyridine (MPEP), fenobam and 2-chloro-4-((2,5-dimethyl-1-(4-(trifluoromethoxy)phenyl)-1H-imidazol-4-yl)ethynyl)pyridine (CTEP), the use of mGluR5 antagonists remains a primary treatment option for FXS (Porter et al., 2005; Yan et al., 2005; Lindemann et al., 2011). However, additional attempts at specific targeting of these receptors have been problematic. Despite mixed success, the development of the mGluR5 antagonist Mavoglurant (AFQ056) has recently been discontinued (April 2014) due to a failure to show improvement over placebo-controlled trials.

Nevertheless, other synaptic proteins have also been involved in the pathology of the syndrome. For instance, loss of FMRP leads to impairments in NMDA receptor-dependent synaptic plasticity in the dentate gyrus (DG), but not in the cornu ammonis area 1 (CA1) subregion (Bostrom et al., 2013), suggesting that functional expression of proteins could be region or even synapse-specific. Additionally, astroglial cells may potentially contribute to enhanced neuronal excitability observed in the mouse model of FXS due to a reduced uptake of glutamate (Higashimori et al., 2013).

On the other hand, we have to account for the excitatory stream counterpart, inhibition, and how this balances circuit activity. Several components of the GABAergic system are also regulated by FMRP expression (reviewed in Paluszkiewicz et al., 2011a). While there is evidence that GABA_A_ receptor subunits show enhanced surface expression such as the γ_2_ subunit (Liu et al., 2013), most other studies suggest the contrary, showing that mRNA expression of α_1_, α_3_ and α_4_β_1_ and β_2_, and γ_1_ and γ_2_, and δ GABA_A_ receptor subunits in the hippocampus (D’Hulst et al., 2006) and the δ subunit in neocortex (Gantois et al., 2006) are down regulated in *Fmr1* KO mice. Further evidence shows that FMRP binds δ subunit mRNA, suggesting a direct influence of FMRP on the expression of δ subunits (Gantois et al., 2006). This latter study supports the hypothesis that tonic inhibition, which is partially mediated by δ subunit containing GABA_A_ receptors, is also down-regulated, contributing to hyperexcitability abnormalities in the neuronal networks of *Fmr1* KO mice (Gantois et al., 2006; Olmos-Serrano et al., 2010; Martin et al., 2014). Thus, GABAergic tonic inhibition has been also taken as a potential candidate for therapeutic treatment in FXS (Olmos-Serrano et al., 2010, 2011; Heulens et al., 2012; Martin et al., 2014).

Despite this information on excitatory/inhibitory balance abnormalities in FXS, an important contributor to the balance has been neglected in these studies: the functional and anatomically diverse population of inhibitory interneurons. Although there is information on how GABA_A_ receptors are affected by the lack of FMRP, few studies address dysfunction of specific presynaptic inhibitory interneurons in FXS. Here we want to summarize some of these studies and discuss how the specific functional properties of different subclasses of inhibitory interneurons are relevant to the study of FXS.

## The contribution of inhibitory interneurons to the FXS phenotype

**Figure 1:**
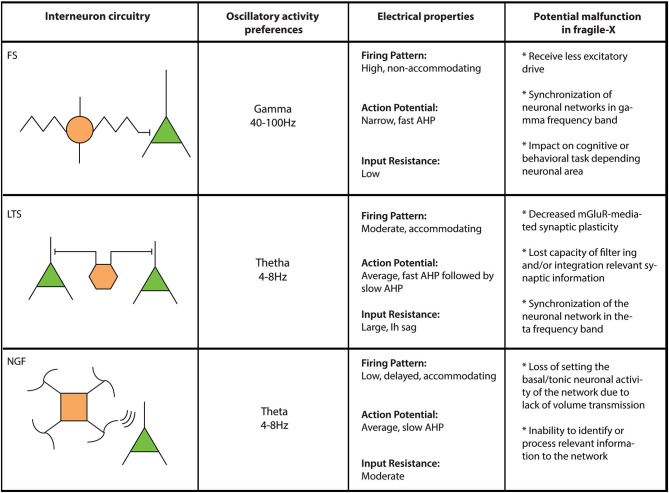
**Comparative table for interneuron populations in FXS**. Three different interneuron types (FS: Fast spiking; LTS: Low threshold spiking and NGF: Neurogliaform cells) are compared here regarding their circuitry/connectivity (left panel), oscillatory preferences (left middle panel), electrical properties (right middle panel) and what their failure would represent in FXS (right panel).

Although often overlooked, the importance of local circuit inhibitory interneurons has rapidly gained attention thanks to a number of studies that have provided essential electrophysiological, anatomical and synaptic insight into the function and role(s) played by this large and heterogeneous cell population (Buzsáki et al., 1992; Gulyás et al., 1993a,b; Buhl et al., 1994; Miles et al., 1996; Gupta et al., 2000; Markram et al., 2004). At the most basic level, interneurons are considered to provide inhibitory control over the excitatory flow of the neuronal network. Their physiological properties and connectivity allow them to control the rhythmic output of large populations of excitatory principal cells as well as other populations of inhibitory interneurons (Cobb et al., 1995; Freund and Katona, 2007; Klausberger and Somogyi, 2008). Interneuronal physiological responses *in vivo* often occur in a time-locked form, discharging in the same temporal window of their preferential oscillatory frequency, suggesting their direct involvement in the synchronization and control of pyramidal cells firing (Klausberger and Somogyi, 2008). Thus, it is possible that interneuron subtypes show a differential participation in the FXS phenotype and likely contribute to specific pathophysiological properties of the neuronal networks where they are involved (Figure 1).

As earlier stated, cortical networks in FXS are hyperexcitable and highly synchronous (Gonçalves et al., 2013; Rotschafer and Razak, 2013). This could explain state-dependent network defects related to intellectual disability, increased incidence of seizures and sensory integration dysfunctions associated with FXS (reviewed in Musumeci et al., 1999; Hagerman and Stafstrom, 2009; Hagerman et al., 2009). Based on heterogeneous anatomy and function of inhibitory interneurons it is likely that inhibitory circuits play important roles in this phenotype. For example, both perisomatic and dendritic-targeting interneurons are known to be involved in the hyperexcitability of the network. Perisomatic interneurons mainly control pyramidal cell excitability by regulating Na+-dependent action potential initiation (Freund and Katona, 2007). In contrast, inhibition arriving at dendritic locations likely have little influence over somal action potential generation but strongly affect local dendritic integration and regulates dendritic Ca^2+^-dependent spike initiation and/or propagation (Miles et al., 1996). From this point of view, while perisomatic interneurons have a role in the synchronization of network circuits imposing a rhythm, dendritic-targeting cells mainly participate in the propagation of synchronized activity waves throughout the network.

In FXS, EEG recordings show elevated relative theta power and reduced relative upper-alpha power (Van der Molen and Van der Molen, 2013), which can be related to longer UP states seen in the neocortex of *Fmr1* KO mouse model (Gibson et al., 2008; Hays et al., 2011). Indeed, local excitation of fast-spiking (FS) inhibitory interneurons, a perisomatic-targeting interneuron that engage preferably in frequencies between 40–100 Hz (Klausberger et al., 2003), is robustly decreased in neocortex in *Fmr1* KO mice (Selby et al., 2007; Patel et al., 2013), which could explain the decrease in synchrony in gamma frequency (Gibson et al., 2008) of the network (Figure 1). However, these inhibitory deficiencies seem to be mediated by polysynaptic responses through local cortical connections instead of monosynaptic or feed-forward responses mediated by thalamic fiber stimulation (Gibson et al., 2008). This is further explained by Patel et al. (2013). When FMRP is conditionally knocked-out in excitatory or inhibitory presynaptic cells, paired recordings reveal that only excitatory responses in inhibitory FS interneurons were decreased by the loss of FMRP (Patel et al., 2013). On the other hand, low threshold spiking (LTS) interneurons, a dendritic-targeting interneuron that contributes to the synchronization of neuronal networks over a wide range of frequencies, including theta and gamma (Szabadics et al., 2001; Blatow et al., 2003), recently have been proposed to control cortical excitability by contributing to the termination of up states in layer II/III (Fanselow and Connors, 2010). Additionally, as opposed to other interneuron subtypes, LTS interneurons respond robustly to metabotropic glutamate receptor (mGluR) activation (Beierlein et al., 2000; Fanselow et al., 2008; Paluszkiewicz et al., 2011b). This robust activation of LTS interneurons is reduced in *Fmr1* KO mice compared to wild type animals (Paluszkiewicz et al., 2011b). The decreased activation of LTS interneurons in *Fmr1* KO mice reduces inhibitory output which in turn alters the synchronization and spike output of excitatory neuronal networks in layer II/III (Paluszkiewicz et al., 2011b). It is also reported that unitary IPSC amplitude mediated by LTS interneurons is increased in somatosensory cortex of *Fmr1* KO mice (Gibson et al., 2008). The fact that this powerful subpopulation of interneurons are tightly coupled by gap junctions (Beierlein et al., 2000; Deans et al., 2001) provides further evidence that the LTS interneuronal microcircuits likely play a key role in hyperexcitable and network synchrony abnormalities in FXS. Moreover, on a network level, LTS interneurons engage in theta frequency activity during mGluR activation (Fanselow et al., 2008; Bostrom et al., 2013) which would explain elevated theta power in EEG from FXS patients (Van der Molen and Van der Molen, 2013).

There is additional evidence that suggest a role for interneurons in FXS with respect to specific activation via neuromodulators. Inhibitory interneurons have differential response to neuromodulators, among them, acetylcholine muscarinic receptors (Cea-del Rio et al., 2010), nicotinic receptors (Bell et al., 2011), serotonin (Chittajallu et al., 2013) and endocannabinoids (eCB; Glickfeld and Scanziani, 2006; Lee et al., 2010). This suggests that alteration of neuromodulatory mechanisms in FXS could differentially affect interneuron cell types. For instance, loss of FMRP broadly affects the eCB signaling system through local 2-arachidonoyl-sn-glycerol (2AG) diminished production (Maccarrone et al., 2010; Zhang and Alger, 2010), possibly because of impaired mGluR5-dependent 2AG formation (Jung et al., 2012). Thus, defects of eCB production will affect inhibitory processes through depolarization suppression of inhibition (DSI; Lee et al., 2010) and slow self-inhibition (SSI; Bacci et al., 2004) mechanisms, suggesting the participation of different set of interneuron cell types in FXS neuronal network abnormalities, including basket cells and LTS cells in the cortex (Bacci et al., 2004; Lee et al., 2010) and basket cells and Schaffer collateral interneurons in the hippocampus (Glickfeld and Scanziani, 2006; Lee et al., 2010). Also, serotonin receptors are affected in the *Fmr1* KO mouse model (Xu et al., 2012b), which can suggest differential regulation of interneuronal cell types such as oriens-laconosum moleculare (O-LM) interneurons of the hippocampus (Chittajallu et al., 2013). Finally, molecular markers such as neuronal nitric oxide synthetase and calbindin are downregulated in FXS (Real et al., 2011; Xu et al., 2012a; Giráldez-Pérez et al., 2013), which suggest that interneurons such as ivy cells, neurogliaform cells (NGF) and bipolar interneuron populations can be diminished in brain circuits of FXS. From these initial studies in the field it is apparent that both monosynaptic and polysynaptic mechanisms of inhibition likely explain some of the neuropathologies observed in FXS. Therefore, more efforts should be addressed to identify specific interneuron populations participating in this syndrome and their roles on network computing and synaptic communication.

Interestingly, inhibitory neurotransmission dysfunction appears to be region selective. As stated above, studies in the cerebral cortex reveal interneuron specific problems. There is a clear lack of excitatory drive to FS interneurons in layer IV (Gibson et al., 2008) and faulty mGluR-dependent activation of LTS interneurons in layer II/III (Paluszkiewicz et al., 2011b). In contrast, inhibitory dysfunction in the amygdala appears to be a “global” loss of inhibitory drive of both phasic (synaptic) and tonic (extrasynaptic) inhibitory neurotransmission onto excitatory principal neurons (Olmos-Serrano et al., 2010; Vislay et al., 2013; Martin et al., 2014). There is also a lack of immunostaining for the synthetic enzyme for GABA and decreased inhibitory connections in the amygdala (Olmos-Serrano et al., 2010). There are biochemical similarities in interneuronal subtypes in the cortex and amygdala, however, there are unique differences to specific spiking properties of specific subtypes such as the parvalbumin-positive interneurons in the amygdala (Woodruff and Sah, 2007a,b). Whether these regional differences are the result of different developmental and migratory patterns of interneuronal populations has yet to be identified. Therefore, further investigation into specific abnormalities in amygdala interneuronal subtypes will need to be explored in future studies in the Fragile-X amygdala.

In summary, while enhanced excitatory neurotransmission leads to hyperexcitable phenotypes, inhibitory interneurons are not just contributing factors but are likely playing a major role in hyperexcitable, hyperresponsiveness and hypersynchronicity of neuronal networks in FXS (Gibson et al., 2008; Hays et al., 2011; Paluszkiewicz et al., 2011b; Gonçalves et al., 2013; Patel et al., 2013). Principally, somatic and dendritic targeting FS and LTS interneurons seem to be the more relevant cell types in cortical abnormalities (Gibson et al., 2008; Paluszkiewicz et al., 2011b), however we cannot rule out the participation of other cortical interneuron cell types such as chandelier, double bouquet, Martinotti or NGFs. Concurrently, interneurons could operate in a different manner depending on the context in which they are involved, from that point of view it is of high priority to study the role of interneurons in different brain areas than the cortex in order to understand their role in these other neuronal networks. In this regard, studies in the Fragile-X amygdala showed that in conditional KO animals, where FMRP is exclusively expressed in inhibitory interneuron populations, that inhibitory neurotransmission dysfunction is comprised of both presynaptic and postsynaptic components (Vislay et al., 2013); therefore suggesting an important role of interneurons in the development and function of this particular brain region in FXS (Olmos-Serrano et al., 2010; Vislay et al., 2013).

## Concluding remarks

Since many FXS patients also present with one or more features of ASDs, insights gained from studying the monogenic basis of FXS could pave the way to a greater understanding of the role of inhibitory interneurons in autism. At this point most of the evidence for interneuron participation is indirect in terms of neuromodulatory activation and downstream excitatory network activation, but very promising in terms of the relevance of their contribution. Thus, understanding how interneurons participate in neuronal network abnormalities seen in FXS lends to a greater understanding for neurodevelopmental disorders that fall in the autism spectrum.

## Conflict of interest statement

The authors declare that the research was conducted in the absence of any commercial or financial relationships that could be construed as a potential conflict of interest.
